# Comparative Cost Modeling of Out-of-Pocket Costs for External Beam Radiation Therapy Versus Stereotactic Body Radiation Therapy in Metastatic Spine Cancer

**DOI:** 10.7759/cureus.115754

**Published:** 2026-09-03

**Authors:** Victoria Wu, Martha Khlopin, Shearwood McClelland III

**Affiliations:** 1 Department of Radiation Oncology, Case Western Reserve University School of Medicine, Cleveland, USA; 2 Department of Radiation Oncology/Department of Neurological Surgery, University of Oklahoma Health Sciences Center, Oklahoma City, USA; 3 Department of Radiation Oncology/Department of Neurological Surgery, University of Oklahoma College of Medicine, Oklahoma City, USA

**Keywords:** ebrt, financial toxicity, medicaid, medicare, metastatic spinal cancer, radiation therapy, sbrt

## Abstract

Purpose: The choice between stereotactic body radiation therapy (SBRT) and external beam radiation therapy (EBRT) for spine metastases depends on several factors: tumor size, tumor location, patient health, prognosis, other treatments (i.e., surgery, minimally invasive procedures, systemic therapy, and supportive care), and treatment goals. Limited evidence exists on insurance's influence on out-of-pocket (OOP) expenses across radiation modalities. This study assesses costs across various insurance plans, including original Medicare and Medicaid.

Methods and materials: National Comprehensive Cancer Network (NCCN) treatment guidelines for symptomatic metastatic spine cancer were used. OOP costs (not adjusted for inflation) were calculated using deductibles, treatment costs, and copays across three public insurance plans-Original Medicare, Medigap Plan G, and Medicaid-over a two-year period for treatments provided in an Ohio hospital. EBRT was defined as one fraction (8 Gray (Gy) x 1), five fractions (4 Gy x 5), or ten fractions (3 Gy x 10). SBRT was defined as one fraction (16-24 Gy x 1), two fractions (24 Gy x 2), three fractions (24-30 Gy x3), four fractions (26-32 Gy x 4), and five fractions (30-40 Gy x5).

Results: Medicare beneficiaries with Original Medicare face OOP costs: $807.01, $871.51, and $961.09 for EBRT one fraction, five fractions, and ten fractions, respectively, after two years. Medicare beneficiaries face charges: $1,127.03, $1,444.92, $1,762.82, $2,398.60, and $2,398.60 for SBRT 1, 2, 3, 4, and 5 fractions, respectively. Under Medigap Part G, patients are responsible only for the annual deductible of $240 (2024), facing a total charge of $480 after two years, regardless of radiation modality and fractionation. Medicaid beneficiaries face no OOP expenses as all expenses are covered with no cap, regardless of treatment option. Under Original Medicare plans, beneficiaries face an OOP cost of 20% (for treatment covered under Part B) of the approved claims charges after the annual $240 deductible is met.

Conclusions: SBRT has higher out-of-pocket costs than EBRT for metastatic spine cancer, depending on the fractionation schedule. These findings highlight the need for strategies to reduce financial toxicity in cancer care.

## Introduction

Spinal metastases remain the most common malignancies of the spine, with the most common primary malignancies arising from breast, lung, prostate, renal, gastrointestinal, and thyroid [1]. Approximately 30-70% of patients with metastatic cancer develop spine metastases [1-3]. Traditionally, external beam radiation therapy (EBRT) has been used for treatment, typically administered over one to two weeks, but its long-term effectiveness in pain control has been limited. As advancements in cancer care extend patient lifespans, the need for more long-lasting and effective treatments has increasingly grown. In response, stereotactic body radiation therapy (SBRT) has become increasingly popular over the past two decades [4-6]. SBRT offers significant advantages over EBRT by delivering higher tumor-targeted doses while minimizing exposure to surrounding healthy tissues.

A multicenter randomized trial has shown that SBRT offers superior and longer-lasting pain control for metastatic spine cancer compared to EBRT. This recent Phase II/III trial (CCTG SC.24/TROG 17.06), involving 229 patients over five years, demonstrated that for patients with symptomatic metastatic spine disease affecting no more than three contiguous levels, SBRT (24 gray (Gy) in 2 fractions) provided significantly better pain relief compared to EBRT (20 Gy in 5 fractions) [4,7]. Early results from a United States (US) series have confirmed the safety and efficacy of 2-fraction spine SBRT [6]. There are varying fractionation regimens for SBRT in the US, ranging from one-fraction to five-fraction to balance safety and effectiveness. Each regimen's choice depends on factors such as tumor size, location, patient condition, and prior treatments.

Despite these theoretical advantages, questions remain about the comparative effectiveness of SBRT versus EBRT in terms of long-term outcomes, safety, and cost-effectiveness, necessitating further investigation into their respective roles in the management of metastatic spine cancer. In addition to clinical outcomes, treatment-related costs are an increasingly important consideration, as out-of-pocket expenses can contribute substantially to financial toxicity among patients with cancer. This study aims to compare the out-of-pocket costs of EBRT versus SBRT for metastatic spine cancer, as well as the financial impact of different SBRT fractionation regimens. This article was previously presented as a meeting abstract at the 2024 American Society for Radiation Oncology 66th Annual Meeting on September 30, 2024, in Washington, DC.

## Materials and methods

Data source and variables

Based on the guidelines of the National Comprehensive Cancer Network (NCCN), guideline-concordant care was identified for the treatment of metastatic spine cancer; for this analysis, the thoracic spine was the anatomic site of metastatic disease [8]. EBRT was defined as one fraction (8 Gray (Gy) x 1), five fractions (4 Gy x 5), or ten fractions (3 Gy x 10). SBRT was defined as one fraction (16-24 Gy x 1), two fractions (24 Gy x 2), three fractions (24-30 Gy x3), four fractions (26-32 Gy x 4), and five fractions (30-40 Gy x5). Treatments and procedures associated with each radiation regimen were identified using Current Procedural Terminology (CPT) and Healthcare Common Procedure Coding System (HCPCS) codes, with the number of billable units determined according to the treatment course and standard billing practices (Table 1). Out-of-pocket costs, including deductibles and copays/coinsurance, were calculated for Medicaid, Original Medicare, Medigap Plan G, and Medicare Part D based on each plan’s specific coverage details [9,10]. Pricing information was sourced from Medicare.gov, medicaid.ohio.gov, aarpmedicareplans.com, and the CMS physician fee schedule as of January 24, 2024 [11-13]. These estimates reflect actual costs rather than projections from claims data, with all procedures performed in an Ohio hospital setting.

**Table 1:** Overview of treatment charges, billing units, and coding EBRT: External Beam Radiation Therapy; SBRT: Stereotactic Body Radiation Therapy; MRI: Magnetic Resonance Imaging; CT: Computed Tomography; OOP: Out of Pocket ^1^Once every five treatments; ^2^Typically matches the number of beams +1; ^3^Daily Cone Beam CT is bundled with SBRT treatment charge.

	Units								CPT Code
	EBRT 1 Fraction	EBRT 5 Fractions	EBRT 10 Fractions	SBRT 1 Fraction	SBRT 2 Fractions	SBRT 3 Fractions	SBRT 4 Fractions	SBRT 5 Fractions	
EBRT									
Radiation Oncologist Initial Office Visit (Complex Consultation)	1	1	1	-	-	-	-	-	CPT: 99205
Physician Treatment Planning Notes	1	1	1	-	-	-	-	-	CPT: 77263
Thoracic Spine MRI w/ and w/o contrast	1	1	1	-	-	-	-	-	CPT: 77014
Complex Teletherapy Isodose Plan	1	1	1	-	-	-	-	-	CPT: 77307
Complex Treatment Device Design and Construction (Facemask)	3	3	3	-	-	-	-	-	CPT: 77334
Complex Planning Simulation	1	1	1	-	-	-	-	-	CPT: 77290
Simple Simulation of Treatment Area (Verification Sim Set Up)	2	2	2	-	-	-	-	-	CPT: 77280
Radiation Treatment Delivery	1	5	10	-	-	-	-	-	CPT: 77412
On-treatment Office Visit with Radiation Oncologist^1^	1	1	2	-	-	-	-	-	CPT: 77427
Continuing medical radiation physics consultation	1	1	1	-	-	-	-	-	CPT: 77336
SBRT									
Radiation Oncologist Initial Office Visit (Complex Consultation)	-	-	-	1	1	1	1	1	CPT: 99205
Physician Treatment Planning Notes	-	-	-	1	1	1	1	1	CPT: 77263
Thoracic Spine MRI w/ and w/o contrast	-	-	-	1	1	1	1	1	CPT: 77014
Thoracic Myelogram for SBRT Planning and Spinal Cord Delineation	-	-	-	1	1	1	1	1	CPT: 62303
Dosimetrist Calculations	-	-	-	1	2	3	4	5	CPT: 77300
Complex Treatment Device Design and Construction (SBRT vaclok)^2^	-	-	-	2	3	4	5	6	CPT: 77334
Special medical radiation physics consultation (for Fusion of CT simulation image with MRI)	-	-	-	1	1	1	1	1	CPT: 77370
Complex Planning Simulation	-	-	-	1	1	1	1	1	CPT: 77290
Verification Simulation	-	-	-	1	1	1	1	1	CPT: 77280
SBRT Treatment (per fraction) 1 or more lesions not to exceed 5 fractions	-	-	-	1	2	3	4	5	CPT: 77373
SBRT Treatment Management (spinal radiosurgery [one to five fractions])	-	-	-	1	2	3	4	5	CPT: 77435
Daily Cone Beam CT^3^	-	-	-	0	0	0	0	0	CPT: 77014
On-treatment Office Visit with Radiation Oncologist^1^	-	-	-	1	1	1	1	1	CPT: 77427
Continuing medical radiation physics consultation	-	-	-	1	1	1	1	1	CPT: 77336
Surveillance/Follow up									
Thoracic Spine MRI w/ and w/o contrast	Every 3 months								CPT: 77014
Radiation Oncology	Every 3 months								CPT: 99214

Ohio was selected as the reference state because several study authors are based in Ohio and have greater familiarity with the state’s Medicaid reimbursement structure and coverage policies, facilitating accurate identification and interpretation of publicly available reimbursement data. Using a single state also provided a consistent geographic and reimbursement framework across treatment pathways. Because Medicare reimbursement may vary geographically and Medicaid reimbursement and cost-sharing policies are state-specific, these estimates should be interpreted as representative of the modeled Ohio setting rather than exact national costs.

Types of insurance plans

Patient OOP responsibility was calculated by applying the deductible, copayment, and/or coinsurance requirements specific to Medicaid, Original Medicare, Medigap Plan G, and Medicare Part D to the estimated reimbursement for each billable service. Original Medicare is a federal health insurance program serving individuals aged 65 and older, as well as younger individuals with qualifying disabilities. It consists of two parts: Part A, which covers hospital-related expenses, and Part B, which provides coverage for medical services [14]. In 2023, Medicare Part B requires beneficiaries to meet an annual deductible of $240, after which they are responsible for 20% coinsurance, with no maximum out-of-pocket limit [15]. To offset these out-of-pocket expenses-such as copayments, coinsurance, and deductibles-beneficiaries may opt for Medicare Supplement plans, commonly known as Medigap plans [16]. Prescription medications obtained from pharmacies are not covered under Original Medicare or Medigap plans. For drug coverage, beneficiaries must enroll in a separate Medicare Part D plan [15,16]. Medicaid, a state-administered program for individuals with limited financial resources and may be required to meet other state requirements. Each state’s Medicaid program contracts with private insurance companies with a Medicare contract to provide coverage for prescription medications under Medicare Part D. As of 2023, 34.1 million beneficiaries were enrolled in Original Medicare (Parts A and B), including 5.3 million with Medigap Plan G, while 22.6 million had standalone Medicare Part D prescription drug plans and 96 million were enrolled in Medicaid [17,18].

## Results

Treatment codes and designations

For both EBRT and SBRT, treatment charges include radiation oncology initial consultation and on-treatment visits, treatment planning notes, thoracic spine MRI, complex planning simulation, dosimetrist calculations, complex treatment device design and construction, medical radiation physics consultation, verification simulation, and radiation treatment delivery. Treatment charges unique to SBRT include thoracic myelogram for SBRT planning and spinal cord delineation, special medical radiation physics consultation, and daily cone beam CT imaging (Table 1). Other costs include surveillance thoracic spine MRI and radiation oncology follow-up every three months after treatment.

Out-of-pocket expenses

Original Medicare enrollees are responsible for paying 20% of Medicare Part B expenses OOP for approved procedures after meeting the deductible, with no cost cap on these costs. Over two years, OOP expenses for EBRT vary by treatment frequency: $807.01 for one fraction, $871.51 for five fractions, and $961.09 for ten fractions. In comparison, costs for SBRT increase with the number of fractions, amounting to $1,127.03 for one fraction, $1,444.92 for two, $1,762.82 for three, $2,080.71 for four, and $2,398.60 for five fractions (Table 2). For individuals with Medigap Plan G, OOP expenses are approximately $480 for both EBRT and SBRT, based on an annual $240 Medicare Part B deductible, assuming it remains constant and is met yearly. Assuming treatment approval, Medicaid beneficiaries have no OOP costs for either treatment option, as all related expenses are fully covered (Table 2).

**Table 2:** Total end-of-year out-of-pocket costs and independent out-of-pocket (OOP) costs for EBRT (1, 5, 10 fractions) and SBRT (1, 2, 3, 4, 5 fractions) EBRT: External Beam Radiation Therapy; SBRT: Stereotactic Body Radiation Therapy; OOP: Out of Pocket.

	Insurance Plan		
	Original Medicare with Medicare Part D	Medigap Plan G with Medicare Part D	Medicaid
EBRT			
Total 2-year OOP Cost for EBRT 1 fraction	$807.01	$480	$0.00
Total 2-year OOP Cost for EBRT 5 fractions	$871.51	$480	$0.00
Total 2-year OOP Cost for EBRT 10 fractions	$961.09	$480	$0.00
SBRT			
Total 2-year OOP Cost for SBRT 1 fraction	$1,127.03	$480	$0.00
Total 2-year OOP Cost for SBRT 2 fractions	$1,444.92	$480	$0.00
Total 2-year OOP Cost for SBRT 3 fractions	$1,762.82	$480	$0.00
Total 2-year OOP Cost for SBRT 4 fractions	$2,080.71	$480	$0.00
Total 2-year OOP Cost for SBRT 5 fractions	$2,398.60	$480	$0.00

## Discussion

Our economic evaluation of metastatic spine cancer radiation treatment from the patient perspective suggests that EBRT costs remain lower than SBRT costs, regardless of fractionation, under original Medicare plans. With increasing fractionation, the cost of treatment increased for both EBRT and SBRT. The two-year OOP cost for EBRT was $807.01 for one fraction, $871.51 for five fractions, and $961.09 for ten fractions. SBRT costs also increased with fractions: $1,127.03 for one, up to $2,398.60 for five fractions. Individuals with Medigap Plan G incur about $480 in OOP costs for both EBRT and SBRT, based on a $240 deductible, while Medicaid beneficiaries have no OOP costs for either treatment, as all related expenses are fully covered.

Our study finds that SBRT, regardless of the fractionation schedule, poses a higher OOP burden than EBRT, with costs increasing alongside the number of fractions. Single-fraction SBRT incurs the least financial burden yet remains substantially more costly than EBRT, where even a 10-fraction regimen is associated with lower OOP expenses than any SBRT schedule. For EBRT, single-fraction treatment has the lowest financial toxicity, at 19% less than a 10-fraction regimen. Clinically, SBRT has shown superior local control and pain relief over conventional EBRT, with both single-fraction (16-24 Gy in 1 fraction) and multi-fraction (e.g., 24 Gy in 2 fractions) schemes commonly used [7,19]. The higher financial burden associated with SBRT may have meaningful clinical implications for patients with spinal metastases, potentially influencing treatment decision-making and access to care, particularly in patients with limited prognosis (< 6 months). A recent cost-effectiveness analysis found that single-fraction SBRT (24 Gy) was more cost-effective than conventional EBRT, but EBRT continued to remain cost-effective for patients who had poor expected survival outcomes; furthermore, the 39% risk of radiation-induced spine fracture from 24 Gy in 1 fraction has led most radiation oncologists to seek fractionation regimens with fracture risk consistent with the 14-17% range commonly observed [7,19,20]. For patients unlikely to benefit from SBRT’s durability advantages, lower OOP costs associated with EBRT may represent an additional consideration in shared decision-making when both treatment approaches are clinically appropriate [20].

In SBRT, the choice of fractionation significantly affects OOP costs, with a five-fraction regimen nearly doubling the financial burden compared to a single-fraction regimen and increasing notably with each additional fraction. Selecting the optimal fractionation for metastatic spine cases requires balancing clinical efficacy, toxicity, patient convenience, and cost. Single-fraction SBRT offers higher local control but may slightly increase the risk of vertebral compression fractures, particularly once doses reach 20 Gy or higher [21-23]. It also provides greater convenience by reducing hospital visits, an advantage for patients with limited life expectancy or severe pain. Although single-fraction SBRT has a higher initial cost than single-fraction EBRT, it may be more cost-effective for patients with more prolonged survival due to improved pain control and reduced need for retreatment. A cost-effectiveness analysis found that SBRT becomes viable at a $100,000 per quality-adjusted life year (QALY) threshold if median survival is at least 11 months, with single-fraction SBRT proving more resource-efficient than multi-fraction regimens [24]. Overall, the decision to minimize fractions in SBRT can reduce financial toxicity when clinically appropriate, supporting findings from previous studies [19,25]. The substantial increase in OOP costs with additional fractions highlights the importance of factoring both clinical and financial considerations into treatment planning. This insight can guide patients and providers in making well-informed decisions, weighing SBRT’s clinical benefits against its financial demands.

Our study is not without limitations. Firstly, our cost estimates are derived from reimbursement data from a single state and may not fully reflect geographic variation or actual patient claims. Additionally, although our analysis evaluates financial burden across multiple public insurance plans using guideline-concordant treatment pathways, privately insured patients were not included, which may limit generalizability to the broader insured population. Nonetheless, this focus on Medicare provides a strong foundation for assessing financial toxicity in an older population often facing metastatic disease, reflecting broader cost challenges that many patients encounter. Thirdly, our analysis is limited to a two-year period, leaving longer-term financial impacts and indirect costs, such as lost income, unexplored; however, given the median survival of spine metastases patients is less than two years, we believe this time period of analysis was reasonable. Lastly, while we include a range of fractionation schedules, factors such as treatment setting, geographic variation, and individual patient choices that might affect costs were not fully accounted for. Despite these limitations, our study offers a thorough examination of out-of-pocket costs across treatment options, yielding valuable insights into the financial burdens associated with radiation therapy. Our analysis includes a comprehensive examination of treatment options, fractionation schedules, and OOP costs over a meaningful two-year period. This timeframe provides insight into both immediate and ongoing financial impacts, offering a useful benchmark for assessing treatment-related financial burdens [26,27].

## Conclusions

SBRT remains a more expensive treatment option than EBRT, regardless of fraction count. Under Medicare, single-fraction EBRT has 19% lower out-of-pocket financial toxicity than 10-fraction EBRT, 40% lower than single-fraction SBRT, 79% lower than two-fraction SBRT, and 197% lower than five-fraction SBRT at two years post-treatment. In spine SBRT, the single-fraction approach has the lowest OOP cost under Medicare at two years post-treatment, while the five-fraction approach has the highest OOP cost: 113% higher than single-fraction, 66% higher than two-fraction, and 36% higher than three-fraction. Even the commonly used three-fraction regimen is 56% and 22% higher in OOP cost than the single and two-fraction options, respectively. Importantly, for the two-fraction regimen, the only one supported by Level 1 evidence, reducing to a single fraction lowers OOP costs by 28% at two years post-treatment. These findings reinforce that minimizing fractionation in spine SBRT, when feasible, can decrease OOP costs and may improve parts of patients' quality of life; this is currently being assessed in the ongoing multicenter Spine Patient Optimal Radiosurgery Treatment for Symptomatic Metastatic Neoplasms (SPORTSMEN) Phase II randomized trial. For patients with spinal metastases, treatment selection should remain primarily guided by clinical appropriateness. However, when EBRT and SBRT are both clinically appropriate, differences in OOP costs may serve as an additional consideration in shared decision-making. By understanding these cost differences, patients and healthcare providers can collaborate to select the most suitable treatment option, considering both clinical and financial factors. 
